# *ACTN4* p.Ile150Met Causes FSGS With Validation in Primary Fibroblasts and Immortalized Podocytes

**DOI:** 10.1016/j.ekir.2025.11.030

**Published:** 2025-11-29

**Authors:** Melanie Grosch, Jan René Haak, Cathiana Kolb, Izabela Plogmann, Mara Sanches Guaragna, Antje Wiesener, Francesca Pasutto, Florian J. Wopperer, Lena Pollinger, Felix B. Engel, Michael S. Wiesener, Mario Schiffer, Tilman Jobst-Schwan

**Affiliations:** 1 Department of Nephrology and Hypertension, Friedrich-Alexander-Universität Erlangen-Nürnberg (FAU), Erlangen, Germany; 2 Department of Medical Genetics and Genomic Medicine, Faculty of Medical Sciences, State University of Campinas, Campinas, Brazil; 3 Institute of Human Genetics, Friedrich-Alexander-Universität Erlangen-Nürnberg (FAU), Erlangen, Germany; 4 Research Centre On Rare Kidney Diseases, Friedrich-Alexander-Universität Erlangen-Nürnberg (FAU), Erlangen, Germany; 5 Department of Nephrology, Center for Rare and Genetic Kidney Diseases, TUM University Hospital rechts der Isar, TUM School of Medicine and Health, Munich, Germany

**Keywords:** genetics, proteinuria, SRNS

## Introduction

Podocytes are highly specialized, terminally differentiated epithelial cells forming an essential part of the glomerular filtration barrier. Through foot processes, they surround capillaries and maintain the selective permeability of the glomerular filter. This architecture relies on a tightly regulated actin cytoskeletonS1; dysregulation causes podocyte injury, detachment, and progression to end-stage renal disease. Multiple actin-regulating proteins, when mutated, are known to cause podocytopathies.^1^ The α-actinin (ACTN) family comprises 4 actin filament–crosslinking proteins.S2^,^S3 ACTN4 (OMIM: 604638S4) is the predominant paralogue in podocytes,S5 where it preserves cytoskeletal integrity.^2^^,^S6 Pathogenic *ACTN4* variants cause podocyte injury and focal segmental glomerulosclerosis (FSGS1, OMIM: 603278S4).^3^, ^4^, ^5^, ^6^^,^S7^,^S8 Reported mutations cluster within the actin-binding domain, increasing actin affinity, and generating actin/ACTN4 aggregates^4^ (Supplementary Table S1 and Figure S1). Functional validation of *ACTN4* variants has typically relied on transient or stable overexpression in immortalized podocytes.

Human dermal fibroblasts (HDFs) provide an alternative; they are obtained by skin biopsy, retain the patient’s genetic background, and have supported the functional validation of novel renal disease genes.S9–S11 However, HDFs have not been applied to the functional assessment of *ACTN4* variants.

Here, we identified a novel *ACTN4* variant, *NC_000019.10: g.38704986C>G*, c.450C>G, p.Ile150Met (NM_004924.6). We show that patient-derived HDFs reproduce the key pathogenic features of *ACTN4* variants—abnormal actin binding, aggregate formation, reduced protein stability, and impaired motility—supporting their utility for functional characterization of novel *ACTN4* variants. All material and methods used are described in detail in the Supplementary Material and Methods.

## Results

### Clinical and Genetic Findings

**Figure 1:**
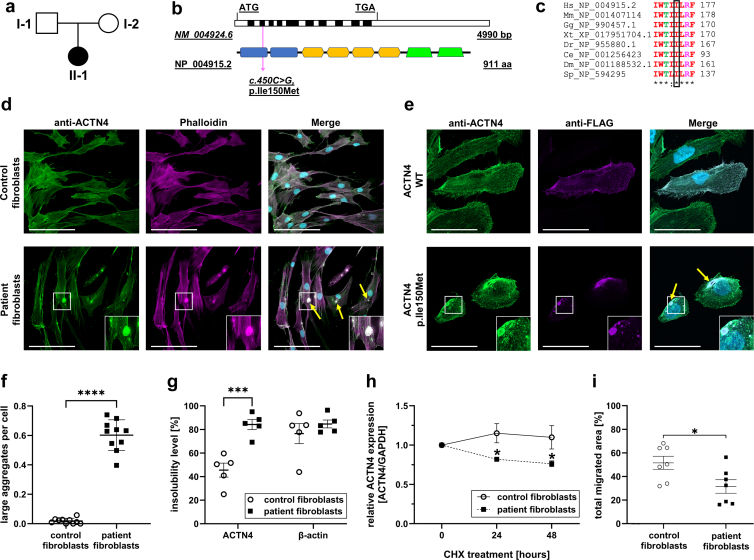
Genetic analysis of the patient with the *ACTN4* c.450C>G, p.Ile150Met variant and functional analysis in cell culture experiments. (a) Pedigree of the index patient. A novel variant in *ACTN4* was detected by whole exome sequencing. Sanger sequencing confirmed absence of the variant in the patient mother (I-2) and presence in the patient (II-1) at NC_000019.10:*g.38704986*. The patient’s father was not available for genetic testing. (b) Graphical visualization of the mRNA (exons in alternating colors) and the protein structure (blue: calponin homology domains; orange: spectrin repeats; green: EF-hand motif) of ACTN4 and localization of the patient variant in Exon 4/the first CH domain (magenta arrow). (c) Alignment with orthologues of different species shows the high conservation of the affected amino acid residue in ACTN4 (black box). (d) Primary human fibroblasts were stained for ACTN4 (green) and F-actin (phalloidin; magenta). In patient fibroblasts, expressing the ACTN4 p.Ile150Met variant, actin/ACTN4 aggregates are visible (yellow arrows), but not in healthy control fibroblasts (*n* = 3). Scale bars correspond to 100 μm. (e) Immortalized human podocytes were electroporated and transfected with FLAG-tagged *ACTN4 WT* or *ACTN4* c.450C>G variant. After transfection, cells were fixed and stained for FLAG-tag (green) and with phalloidin (magenta) and DAPI (blue). Cell imaging was performed using a Zeiss confocal microscope at 20× magnification. Control transfected cells did not show actin/FLAG-tagged ACTN4 WT aggregates (upper panel). Contrary to that, FLAG-tagged ACTN4 p.Ile150Met forms actin/ACTN4 aggregates (lower panel; yellow arrows), similarly to the patient fibroblasts. Scale bars correspond to 50 μm. (f) Particle analysis script in Fiji ImageJ was used to quantify aggregates in fibroblasts. Each microscopic field of view was investigated for the number of large aggregates (size > 0.97 μm^2^) related to cell numbers (based on nuclei numbers). The graph represents 2 technical replicates per group with 5 microscopic fields of view (20×) per replicate (control: 37 aggregates/1714 nuclei in total, patient: 939 aggregates/1647 nuclei; mean and SEM; ∗∗∗∗*P* < 0.0001). (g) Healthy control fibroblasts and patient fibroblasts were lysed in Triton X-100 containing lysis buffer and centrifuged to obtain Triton X-100 insoluble (TI) and soluble (TS) fractions. TI fractions contain large cytoskeletal structures including F-actin, whereas TS fractions contains G-actin. Densitometry of fractionation experiments showed significantly higher insolubility level of ACTN4 (displayed as relative expression levels TI/[TI+TS]) in patient fibroblasts (ACTN4 p.Ile150Met) compared with control fibroblasts (ACTN4 WT). In contrast, the distribution of actin is comparable between control and patient fibroblasts. (Results of *n* = 5 experiments, mean and SEM, ∗∗∗*P* < 0.001). (h) Control and patient fibroblasts were treated with 100 μg/ml CHX for up to 48 hours. Proteins were isolated and subjected to immunoblotting. Densitometry of CHX pulse chase assay experiments (normalized by GAPDH) displayed a faster degradation of ACTN4 p.Ile150Met (patient fibroblasts) compared with ACTN4 WT (control fibroblasts). Results of *n* = 3 experiments are displayed (mean and SEM, ∗*P* < 0.05). (i) Control and patient fibroblasts were plated in an ibidi 2-chamber silicone insert. Insert was removed and cell migration was monitored in real time. Migration of patient fibroblasts (ACTN p.Ile150Met) is significantly reduced compared with control (ACTN4 WT) fibroblasts. Total migrated area in % was manually outlined and measured using ImageJ. Results of *n* = 7 experiments are displayed (mean and SEM; ∗ *P* < 0.05). CHX, cycloheximide; WT, wild type.

We evaluated a 32-year-old woman with severe childhood-onset steroid-resistant nephrotic syndrome and no family history of renal disease (Figure 1a). Extrarenal manifestations included sensorineural hearing impairment and asymmetric breast development. At age 5, kidney biopsy revealed minimal change glomerulonephritis, progressing to end-stage renal disease by the age of 8 years. Native kidney tissue was unavailable for histological review.

She underwent her first kidney transplant at the age of 10 years, which failed because of recurrent pyelonephritis. A second transplant, from her mother at the age of 31 years was initially complicated by chronic active T-cell–mediated rejection (BANFF IA) after azathioprine substitution for mycophenolate mofetil to enable pregnancy. Graft function stabilized after mycophenolate mofetil reintroduction alongside tacrolimus and prednisolone. Whole-exome sequencing with analysis of the abnormal renal glomerulus morphology panel (HP_0000095, October 2019) identified a heterozygous *ACTN4* c.450C>G, p.(Ile150Met) variant, located in exon 4 within the first calponin-homology domain (Figure 1b). No additional variants appeared in an extended 726-gene abnormal renal physiology panel (HP_0012211, May 2024). Screening of 149 genes associated with hearing impairment (https://hereditaryhearingloss.org) was negative. Only one other *ACTN4* variant (p.Trp59Arg) has been associated with both childhood end-stage renal disease and hearing impairment, suggesting an expanded extrarenal phenotype.S7^,^S12

Segregation analysis showed absence of the variant in the healthy mother (Supplementary Figure S2). Paternal DNA from the father and other relatives was unavailable, but no family history of renal disease was reported. The variant was absent from gnomADS13 and ClinVarS14. Cross-species alignment confirmed conservation of Ile150 across orthologues (Figure 1c, Table 1). *In silico* prediction tools (SIFTS15, MutationTasterS16, PolyPhen-2S17) classified the variant as deleterious.

**Table 1:** Genetic data of a novel *ACTN4* variant

Gene	*ACTN4*
Accession number	NM_004924.6
hg38 position	chr19:38704986
Nucleotide change	c.450C>G
Amino acid change	p.Ile150Met
Exon (zygosity)	4 (heterozygous)
gnomAD	not applicable
ClinVar	not applicable
SIFT	deleterious
Mutation Taster	disease causing
PolyPhen-2	1
ACMG classification	likely pathogenic (PS3, PM1, PM2, PP3)
Amino acid conservation to species	*Schizosaccharomyces pombe*

ACMG, American College of Medical Genetics.

Whole exome sequencing was performed using a blood sample of a female patient with primary focal segmental glomerulosclerosis using a lllumina HiSeq 2500 (Illumina, San Diego, CA). Subsequent analysis using the HP_0000095 gene panel (abnormal renal glomerulus morphology) revealed a heterozygous nucleotide change at position 450 from cytosine to guanine, leading to an amino acid change at position 150 from isoleucine to methionine. The affected amino acid is highly conserved across different species. The amino acid change is predicted to be deleterious and disease-causing by different algorithms (SIFTS15 MutationTasterS16 PolyPhen-2S17). The variant is not present in gnomAD or in ClinVar.

ACMG classificationS24: PS3: well-established *in vitro* or *in vivo* functional studies supportive of a damaging effect on the gene or gene product; PM1: located in a mutational hot spot and/or critical and well-established functional domain (e.g., active site of an enzyme) without benign variation; PM2: absent from controls in Exome Sequencing Project, 1000 Genomes Project, or Exome Aggregation Consortium; PP3: multiple lines of computational evidence support a deleterious effect on the gene or gene product (conservation, evolutionary, splicing impact, etc.).

### ACTN4 p.Ile150Met Forms Actin/ACTN4 Aggregates in Primary Fibroblasts and Immortalized Podocytes

To assess cellular effects, we established HDFs from the patient and an age- and sex-matched control. HDFs are well-suited for ACTN4 functional studies because they express ACTN4 robustly^7^ and circumvent limitations of podocyte culture.S18

In control fibroblasts, ACTN4 colocalized with filamentous actin (Figure 1d). In patient fibroblasts, actin/ACTN4 aggregates were observed (Figure 1d and f). For validation, we transfected immortalized human podocytes with FLAG-tagged ACTN4 wild type or ACTN4 p.Ile150Met. Aggregates occurred only in cells expressing ACTN4 p.Ile150Met (Figure 1e). To confirm aggregate formation, we performed actin fractionation of control and patient fibroblasts, separating Triton X-100 insoluble (TI) and soluble (TS) fractions. The TI fraction contains large structures, including cytoskeletal aggregates, whereas soluble components are contained in the TS fraction.^2^^,^S20 In the patient fibroblasts, significantly more ACTN4 was detected in the TI fraction than in the control, whereas no significant differences were observed in the TS fraction (Figure 1g, Supplementary Figure S2). Actin distribution between TI and TS fractions did not differ between groups (Figure 1g, Supplementary Figure S3). These findings suggest increased actin affinity of ACTN4 p.Ile150Met in both patient fibroblasts and transfected podocytes compared with the wild type.

### Reduced Stability of ACTN4 p.Ile150Met

Actin binding is mediated by the tandem N-terminal calponin-homology domains.S21 Variants in these domains reduce ACTN4 stability.^5^ Five computational algorithms predicted destabilization of ACTN4 p.(Ile150Met), consistent with analogous substitutions in ACTN1 and ACTN3 (Supplementary Table S2).

Cycloheximide chase assaysS22 support this prediction; ACTN4 levels in control fibroblasts remained stable over 48 hours, whereas patient fibroblasts showed significant decline after 24 hours and further loss at 48 hours (Figure 1h, Supplementary Figure 4A and B).

### Impaired Fibroblast Motility

ACTN4 mutations forming aggregates are known to impair cell motility.^2^^,^^5^ To assess this, we performed migration assays. At 24 hours, control fibroblasts covered a significantly larger area than patient fibroblasts (Figure 1i, Supplementary Figure 4C/D), indicating impaired motility and cytoskeletal reorganization associated with ACTN4 p.Ile150Met.^7^^,^S23

## Conclusion

Pathogenic *ACTN4* variants were first linked to familial focal segmental glomerulosclerosis 2 decades ago.^3^ Since then, > 20 variants have been described, most within the actin-binding domain. Our patient with early-onset steroid-resistant nephrotic syndrome and end-stage renal disease harbored the novel variant, *ACTN4* c.450C>G; p.Ile150Met. Functional assays demonstrated all major hallmarks of pathogenic *ACTN4* variants, namely aggregate formation, increased actin affinity, reduced protein stability, and impaired cellular motility.

Feng *et al.*^4^ outlined 4 criteria to establish pathogenicity of *ACTN4* variants, namely segregation, localization to a conserved domain, abnormal aggregates, and increased actin binding. Our variant fulfils 3 of these 4; segregation was not possible because of lack of paternal DNA. Under the American College of Medical Genetics criteria,S24 *ACTN4* c.450C>G, p.Ile150Met is likely pathogenic (PS3, PM1, PM2, PP3).

The mechanism by which *ACTN4* variants cause podocyte injury remains incompletely understood. Some variants destabilize the cytoskeleton, whereas others form aggregates that may act through a toxic gain-of-function,^2^^,^^4^^,^^5^^,^^8^^,^S25 Our findings suggest that early-onset variants, such as p.Ile150Met, act through aggregate toxicity, consistent with previous studies on ACTN4 p.Met240Thr.^6^ Analogous to protein aggregation in neurodegenerative diseases such as Huntington’s or Parkinson’s,S26^,^S27 ACTN4 aggregates may disrupt cellular homeostasis.S28

Our study highlights the potential of primary HDFs as a functional assay system. Compared with immortalized podocytes, HDFs are easy to obtain,S29 maintain proliferative capacity for multiple passages,S30 and preserve the native genetic background.^9^ Importantly, we demonstrated hallmark features of pathogenic *ACTN4* variants directly in patient-derived fibroblasts, avoiding immortalization or artificial overexpression.S31 Limitations of our study include donor age effects,S32^,^S33 passage-dependent changes,S34 and absent expression of some disease-relevant genes, though CRISPR-based activation strategies may overcome this.S35 However, reliance on the patient’s genetic background can be a drawback because the absence of an isogenic control line makes it difficult to exclude contributions from other genetic factors.

Previous studies using murine fibroblasts or immortalized human fibroblasts suggested this approach^8^^,^S28^,^S36; here we show, for the first time, that primary HDFs can directly validate a novel *ACTN4* variant. This expands the toolkit for functional genomics of kidney diseases and strengthens the case for using patient-derived fibroblasts in personalized medicine.

Clinically, recognition of pathological *ACTN4* variants is critical. For our patient, the *ACTN4* variant indicates a structural podocytopathy rather than an immune-mediated disease. Knowledge of the genetic etiology could have prevented unnecessary long-term immunosuppression during childhood, reducing risks for infection and malignancy. Furthermore, recurrence of the primary disease in the kidney allograft can be excluded, with important implications for transplantation management.

In conclusion, we identified a novel *ACTN4 c.450>G*, p.Ile150Met variant in a patient with early-onset steroid-resistant nephrotic syndrome. Functional studies in patient-derived fibroblasts and podocytes confirm its deleterious effects on actin binding, protein stability, and cellular motility. Our data establish this variant as likely pathogenic and demonstrate that primary HDFs represent a powerful, accessible *in vitro* model for the characterization of novel *ACTN4* variants. This approach can support genetic diagnosis, inform patient management, and advance personalized medicine in glomerular diseases.

## Disclosure

All the authors declared no competing interests.

## Supplementary Material

Supplementary File (PDF) **Supplementary Methods. Supplementary References. Figure S1.** Location of previously described pathogenic and likely pathogenic *ACTN4* variants present in ClinVar and the novel variant identified in this work. **Figure S2.** Segregation analysis showed absence of the variant in the healthy mother. **Figure S3.** ACTN4 p.(Ile150Met) forms Triton X-100 insoluble structures. **Figure S4.** ACTN4 p.(Ile150Met) is less stable than the wild type form and leads to reduced motility in primary fibroblast. **Table S1.** Previously identified pathogenic and likely pathogenic ACTN4 variants present in ClinVar and the novel variant identified in this work. **Table S2.** ACTN4 p.(Ile150Met) is less stable than the wild type form.

## Footnotes

Supplementary File (PDF)

**Supplementary Methods.**
**Supplementary References**. **Figure S1.** Location of previously described pathogenic and likely pathogenic *ACTN4* variants present in ClinVar and the novel variant identified in this work. **Figure S2.** Segregation analysis showed absence of the variant in the healthy mother. **Figure S3.** ACTN4 p.Ile150Met forms Triton X-100 insoluble structures. **Figure S4.** ACTN4 p.Ile150Met is less stable than the wild type form and leads to reduced motility in primary fibroblast. **Table S1.** Previously identified pathogenic and likely pathogenic ACTN4 variants present in ClinVar and the novel variant identified in this work. **Table S2.** ACTN4 p.Ile150Met is less stable than the wild type form.
