# Supplementary material for: ACTN4 p.Ile150Met Causes FSGS With Validation in Primary Fibroblasts and Immortalized Podocytes
Source: Kidney Int Rep. 2025 Nov 29;11(2):103706. doi: 10.1016/j.ekir.2025.11.030 (PMC12800584; doi:10.1016/j.ekir.2025.11.030)
Supplement: Supplementary File (PDF) — Supplementary Methods. Supplementary References. Figure S1. Location of previously described pathogenic and likely pathogenic ACTN4 variants present in ClinVar and the novel variant identified in this work. Figure S2. Segregation analysis showed absence of the variant in the healthy mother. Figure S3. ACTN4 p.(Ile150Met) forms Triton X-100 insoluble structures. Figure S4. ACTN4 p.(Ile150Met) is less stable than the wild type form and leads to reduced motility in primary fibroblast. Table S1. Previously identified pathogenic and likely pathogenic ACTN4 variants present in ClinVar and the novel variant identified in this work. Table S2. ACTN4 p.(Ile150Met) is less stable than the wild type form. [file mmc1.pdf]

ACTN4 p.Ile150Met causes focal segmental glomerulosclerosis with validation in primary fibroblasts and immortalized podocytes (Grosch et al.)

Supplementary material

- Supplementary Material and Methods
- Supplementary Table 1: Previously identified pathogenic and likely pathogenic ACTN4 variants present in ClinVar and the novel variant identified in this work.
- Supplementary Table 2: ACTN4 p.(Ile150Met) is less stable than the WT form.
- Supplementary Figure 1: Location of previously described pathogenic and likely pathogenic ACTN4 variants present in ClinVar and the novel variant identified in this work.
- Supplementary Figure 2: Segregation analysis showed absence of the variant in the healthy mother.
- Supplementary Figure 3: ACTN4 p.(Ile150Met) forms Triton X-100 insoluble structures.
- Supplementary Figure 4: ACTN4 p.(Ile150Met) is less stable than the WT form and leads to reduced motility in primary fibroblast.
- Supplementary References

Supplementary Material and Methods

Patient and ethical approval

The study was approved by the Ethics Committee of the University Hospital, Erlangen, Germany (protocol no. 251_18 B). Informed consent was obtained from all participants.

Whole exome sequencing (WES)

WES of the index patient´s DNA was performed on an Illumina HiSeq 2500 (Illumina, San Diego, CA). Processing of raw data (alignments and variants calls, subsequent filtering and inspection) was performed as described previously [S38]. WES data was analysed using a panel for variances in 126 genes connected to *abnormal renal glomerulus morphology* (HP_0000095, October 2019) and for variances in 149 genes associated with hereditary hearing loss (https://hereditaryhearingloss.org; October 2019). All relevant variants were confirmed by Sanger sequencing. Prediction of deleterious and disease causing were performed using SIFT [S39], MutationTaster2021 [S40] and Polyphen-2 [4S41] algorithms.

Generation and cultivation of primary fibroblasts

Human dermal fibroblasts (HDF) were generated from sterile skin punch biopsies cultured in DMEM (Dulbecco's Modified Eagle's Medium) supplemented with 10–20% fetal calf serum (FCS; PAN-Biotech, Aidenbach, Germany), 1% sodium pyruvate and 1% penicillin and streptomycin (P/S; Sigma-Aldrich, Merck, St. Louis, MO, USA) in 5% CO_2 at 37°C as described before [S42]. Control fibroblasts were obtained from a healthy age- and sex-matched volunteer. Fibroblasts from passages 5–10 were used for the experiments. For cycloheximide (CHX) chase assay experiments, primary fibroblasts were treated with 100 µg/ml CHX for 24 or 48 hours, respectively. After treatment, cells were lysed in RIPA buffer and used for immunoblotting.

Plasmid generation

Full-length human wildtype (WT) *ACTN4* cDNA was cloned into a pBluescript II SK (+) vector by GenScript (Piscatawy, NJ, USA). Patient variant *ACTN4* c.450C>G was generated using the Q5® Site-directed mutagenesis kit (New England Biolabs, #E0554S, Ipswich, MA, USA) according to the commercial protocol. Recombination of *ACTN4 WT* and variant into a pcDNA3 FLAG HA vector (Addgene; #10792) was performed using NEBuilder® HiFi DNA Assembly (New England Biolabs, #E2621S, Ipswich, MA, USA).

Podocyte cell culture and transfection

Conditionally immortalized human podocytes (kindly provided by Moin Saleem, Children's and Renal Unit and Bristol Renal, University of Bristol) were maintained in RPMI medium 1640 (Gibco, Thermo Fisher Scientific, Waltham, MA, USA) supplemented with 10% heat-inactivated FCS, 1% P/S, and 0.1% insulin-transferrin-selenium (Thermo Fisher Scientific, Waltham, MA, USA) and cultivated at 33°C for proliferation. After thermo-switching to 37°C, cells were differentiated for 10 days before transfection. Prior to transfection, cells were resuspended in Opti-MEM serum-reduced medium (Gibco, Thermo Fisher Scientific, Waltham, MA, USA) and transfected using electroporation in a ProGenetor II (Hoefer Scientific Instruments, Holliston, MA, USA). Two days after transfection, podocytes were prepared for immunofluorescence.

Immunofluorescence

Cells were washed in phosphate buffered saline (PBS), fixed in 4% PFA for ten minutes and permeabilized using 0.5% Triton X-100 in PBS for ten minutes at room temperature. Samples were then washed in PBS and blocked for one hour in blocking buffer (5% donkey serum, 1% bovine serum albumin (BSA) (PAN Biotech, Aidenbach, Germany) in PBS) at room temperature. Afterwards, cells were incubated with rabbit anti-human ACTN4 primary antibody (1:200, Cell Signaling Technology, #15145S, Ipswich, MA, USA) in blocking buffer overnight at 4°C. Thereupon, samples were washed in PBS and incubated with donkey anti-rabbit Alexa Fluor™ Plus 488 coupled secondary antibody (1:1000, Thermo Fisher Scientific, #A32790, Ipswich, MA, USA) and Alexa Fluor™ 647 Phalloidin (1:40, Thermo Fisher Scientific, #A30107, Ipswich, MA, USA) in PBS for one hour. DNA was visualized using DAPI (1:10000, Thermo Fisher Scientific, #D3571, Ipswich, MA, USA). Image acquisition was performed using a Zeiss LSM800 confocal laser scanning microscope with Airyscan module (Carl Zeiss AG, Oberkochen, Germany) and processed using ZEN blue software (Carl Zeiss AG, Oberkochen, Germany) and Fiji [S43].

Aggregate quantification

Fluorescent images with triple staining were generated as described above. The "Analysis particles" script in Fiji [S43] was used to quantify aggregates in fibroblasts using following protocol:

Cell number was assessed by nuclei counting using following protocol in the DAPI staining:

Process>Filter>Gaussian blurs	Sigmaradius 1.5
Image>Adjust>Threshold	70-255
Process>Binary>Watershed	
Analyze>Analyze Particles	Size 200-25000 (pixel units)<br>Circularity 0.1-1.00<br>include holes<br>display results<br>summarize<br>show outlines

ACTN4 aggregates were assessed by particle counting using following protocol:

Image>Adjust>Threshold	150-255
Analyze>Analyze Particles	Size 10-25000 (pixel units)<br>Circularity 0.5-1.00<br>include holes<br>display results<br>summarize<br>show outlines

Each microscopic field of view was investigated for the number of large aggregates (size >0,97 μm^2) related to cell numbers (based on nuclei numbers).

Actin fractionation assay

Actin fractionation assay was performed as described before [5] with small changes. HDFs were lysed in lysis buffer (20 mM Tris-HCl (pH 7.5), 50 mM sodium chloride, 50 mM sodium fluoride, 15 mM sodium pyrophosphate, 2 mM sodium vanadate, 1% Triton X-100 and complete protease inhibitor tablet (Roche, Basel, Switzerland)). To obtain the Triton X-100 insoluble fraction, lysates were centrifuged at 15.000 x *g* for ten minutes at 4°C. The supernatant contains the Triton X-100 soluble (TS) fraction and the pellet the Triton X-100 insoluble (TI) fraction. The TI fraction contains large cytoskeletal aggregates like actin/ACTN4 bundles. TI fraction was further resuspended in RIPA buffer. Protein concentrations of both fractions were determined using DC Protein Assay (Bio-Rad, Watford, UK).

Immunoblotting

Equal amounts of protein were separated by sodium dodecyl sulphate–polyacrylamide gel electrophoresis and transferred to polyvinyl fluoride (PVDF). After blocking in a 5% BSA/TBS-T solution, membranes were incubated with polyclonal rabbit anti-ACTN4

antibody (Cell Signaling, #15145S, Dauvers, MA, USA) overnight at 4°C. Afterwards, membranes were washed with TBS-T and incubated with horseradish peroxidase (HRP)-coupled donkey anti-rabbit antibody (Cell Signaling Technology, #7074-P2, Dauvers, MA, USA). As loading controls, membranes were stained with either monoclonal mouse anti-GAPDH antibody (GeneTex, #GTX100118, Irvine, CA, USA) or polyclonal rabbit anti-actin antibody (Proteintech, #20536-1-AP, Rosemont, IL, USA) overnight at 4°C, followed by incubation with HRP-coupled donkey anti-mouse (Cell Signaling Technology, #7076-P2, Dauvers, MA, USA) or HRP-coupled donkey anti-rabbit antibodies, respectively. Signal detection of bands was performed using an Amersham Imager 600 (GE Healthcare, Amersham, UK). Densitometry was performed using Fiji [S43].

Migration assay

HDFs were seeded in equal numbers into each chamber of a removable cell culture insert (ibidi, #80206, Gräfelfing, Germany). The chambers were divided by a non-diffusible barrier. Cells were allowed to attach for 24 hours. Prior to removing the insert, cells were serum-starved for 24 hours. After removing the insert, cells were immediately placed in a BIOREVO BZ-9000 (Keyence, Osaka, Japan). Pictures were taken once every hour for 24 hours. Migrated area was assessed manually using Fiji [6].

Statistical analysis and visualization

Biological replicates of detected western blot intensities were normalized using the optimal alignment method described in Degasperi *et al.*[S44]. For this, a custom script was written in MATLAB™ 2022a.

Graphic visualization and statistical analysis were performed using GraphPad Prism 10 (GraphPad Software LLC., Boston, MA, USA). Normality distribution was tested using Shapiro-Wilk test. Comparison between groups was performed using one sample t-test, Kruskal-Wallis test or two-way ANOVA. *P* values were defined as followed: * = P <0.05.

Supplementary Table 1: Previously identified pathogenic and likely pathogenic *ACTN4* variants present in ClinVar and the novel variant identified in this work. The novel variant identified in this work is highlighted in yellow. Abbreviations: ABD, actin binding domain; ASBD, asymmetric breast development; CKD5, chronic kidney disease stage 5; FSGS, focal segmental glomerulosclerosis; KTX, kidney transplantation; NBHFHL: non-syndromic bilateral high frequency hearing loss; NS: nephrotic syndrome; SHI: sensorineural hearing impairment.

ClinVar accession	Nucleotide change	Amino acid change	Exon	Variant type	Affected domain	Age of disease onset	Phenotype	ACMG classification	First description
VCV001028308	c.175C>T	p.Trp59Arg	2	missense	ABD	5	FSGS; Recurrent proteinuria after KTX; NBHFHL	Likely pathogenic	Weins, A. *et al.*, 2005 [S7]
VCV004084962	c.214G>C	p.Glu72Gln	2	missense	ABD	5	Not provided	Likely pathogenic	07/2025
VCV002736886	c.445_447del	p.Ile150del	4	Inframe deletion	ABD	20-30	FSGS (incomplete penetrance)	Pathogenic	Weins, A. *et al.*, 2005 [S7]

ClinVar accession	Nucleotide change	Amino acid change	Exon	Variant type	Affected domain	Age of disease onset	Phenotype	ACMG classification	First description
n/a	c.450C>G	p.Ile150Met	4	missense	ABD	5	FSGS; Recurrent proteinuria after KTX; SHI; ASBD	Likely pathogenic	This study
VCV000599130	c.458T>C	p.Phe153Ser	4	missense	ABD	n/a	FSGS	Likely pathogenic	12/2024, clinical testing (Bioscientia Institut fuer Medizinische Diagnostik GmbH, Sonic Healthcare, Germany)
VCV002572429	c.493G>A	p.Ala165Thr	5	missense	ABD	17	NS; CKD5	Likely pathogenic	He, Z. *et al*, 2022 [S45]
VCV003061635	c.506T>G	p.Leu169Arg	5	missense	ABD	n/a	ACTN4-related disorder	Likely pathogenic	12/2023, clinical testing (PreventionGenetics, WI, USA)

ClinVar accession	Nucleotide change	Amino acid change	Exon	Variant type	Affected domain	Age of disease onset	Phenotype	ACMG classification	First description
VCV000599063	c.510_512del	p.Leu171del	5	Inframe deletion	ABD	n/a	FSGS	Likely pathogenic	01/2018, clinical testing (Bioscientia Institut fuer Medizinische Diagnostik GmbH, Sonic Healthcare, Germany)
VCV003068392	c.517T>C	p.Cys173Arg	5	missense	ABD	n/a	FSGS	Likely pathogenic	01/2024 clinical testing (MVZ Medizinische Genetik Mainz, Germany)
VCV000235864	c.584G>A	p.Gly195Asp	6	missense	ABD	13	NS;ESRD	Pathogenic	Bartram, MP. *et al.*, 2016 [5]
VCV002500724	c.584G>T	p.Gly195Val	6	missense	ABD	n/a	FSGS	Pathogenic	02/2022
VCV003035302	c.608A>C	p.His203Pro	6	missense	ABD	n/a	ACTN4-related disorder	Likely pathogenic	01/2025, clinical testing (PreventionGenetics, WI, USA)

ClinVar accession	Nucleotide change	Amino acid change	Exon	Variant type	Affected domain	Age of disease onset	Phenotype	ACMG classification	First description
VCV003236342	c.718A>G	p.Met240Val	7	missense	ABD	4	FSGS	Likely pathogenic	05/2024 clinical testing (MVZ Medizinische Genetik Mainz, Germany)
VCV003900730	c.757G>A	p.Asp253Asn	8	missense	ABD	n/a	FSGS	Likely pathogenic	06/2025 clinical testing (MVZ Medizinische Genetik Mainz, Germany)
VCV000005420	c.763A>G	p.Lys255Glu	8	missense	ABD	n/a	FSGS (high, but not full penetrance)	Pathogenic	Kaplan, JM. *et al.*, 2000 [3]
VCV000005421	c.776C>T	p.Thr259Ile	8	missense	ABD	n/a	FSGS (high, but not full penentrance)	Pathogenic	Kaplan, JM. *et al.*, 2000 [3]
VCV000005422	c.784C>T	p.Ser262Pro	8	missense	ABD	n/a	FSGS (high, but not full penentrance)	Pathogenic	Kaplan, JM. *et al.*, 2000 [3]

Supplementary Table 2: ACTN4 p.(Ile150Met) is less stable than the WT form. Effect prediction of ACTN4 p.(Ile150Met) by different software. Likewise, same change in ACTN1 and ACTN3 is predicted to have also a destabilizing effect.

Platform	PDB: 2R0O (ACTN4)		PDB: 2EYN (ACTN1)		PDB: 1WKU (ACTN3)	
	ΔΔG (kcal/mol)	Effect	ΔΔG (kcal/mol)	Effect	ΔΔG (kcal/mol)	Effect
Cupsat	-1.16	Destabilizing	-0.83	Destabilizing	-0.45	Destabilizing
Eris	>10	Destabilizing	6.61	Destabilizing	5.31	Destabilizing
IMutant 2.0	-0.81	Destabilizing	-0.84	Destabilizing	-0.86	Destabilizing
POPMUSIC	0.7	Destabilizing	0.51	Destabilizing	0.81	Destabilizing
DUET	-0.64	Destabilizing	-0.526	Destabilizing	-0.867	Destabilizing

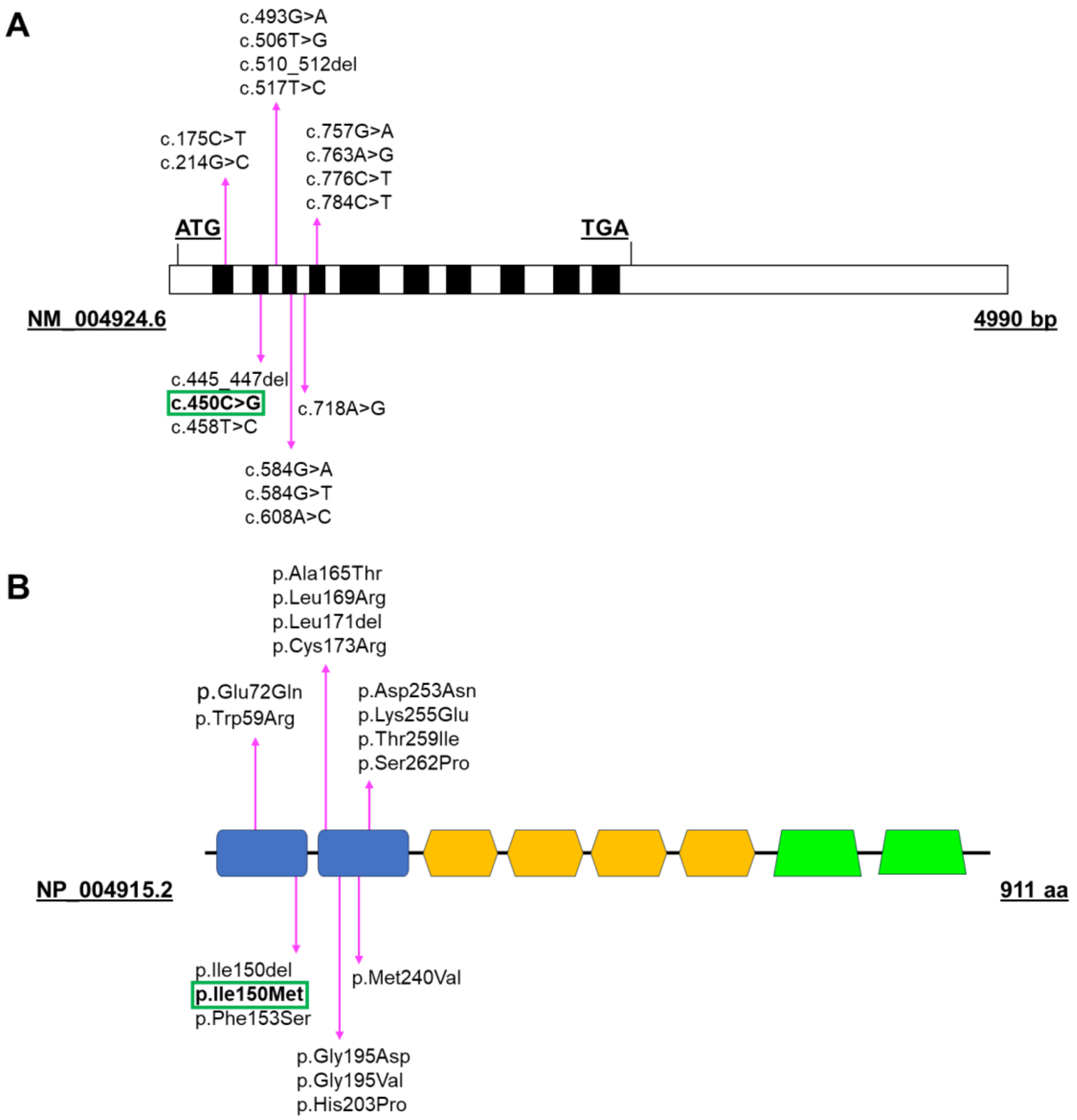

Supplementary Figure 1: Location of previously described pathogenic and likely pathogenic *ACTN4* variants present in ClinVar and the novel variant identified in this work.

Only pathogenic and likely pathogenic variants according to ClinVar are shown. (**A**) Graphical visualization of *ACTN4* mRNA (exons in alternating colors). Location of previously described variants and mutations in their respective exon. The novel variant described in this work (c.450C>G) is in bold and marked with a green square. (**B**) Protein structure of ACTN4 (blue: calponin homology domains; orange: spectrin repeats; green: EF-hand motif) and localization of previously described variants and mutations in their respective domain. The novel variant described in this study (p.Ile150Met) is in bold and marked with a green square.

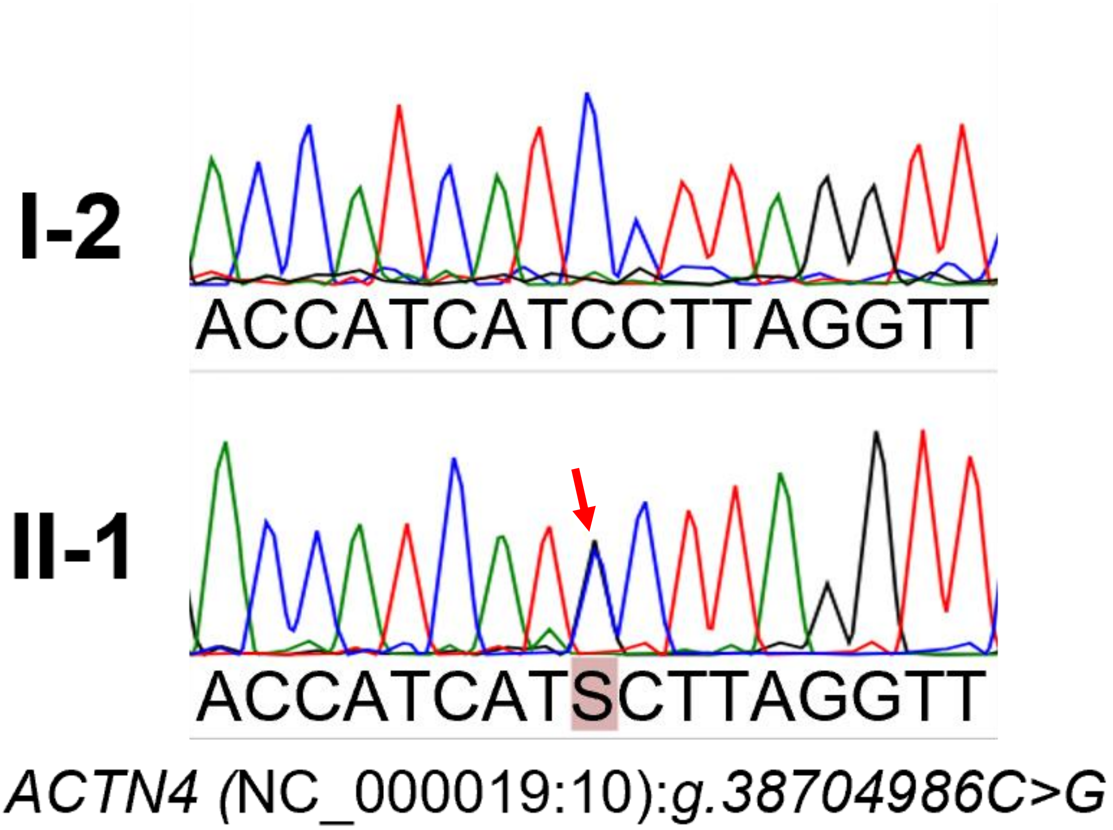

Supplementary Figure 2: Segregation analysis showed absence of the variant in the healthy mother. Sanger sequences of mother (I-2) and affected index patient (II-1). Please be aware of the high overlap of the C and G peak (red arrow).

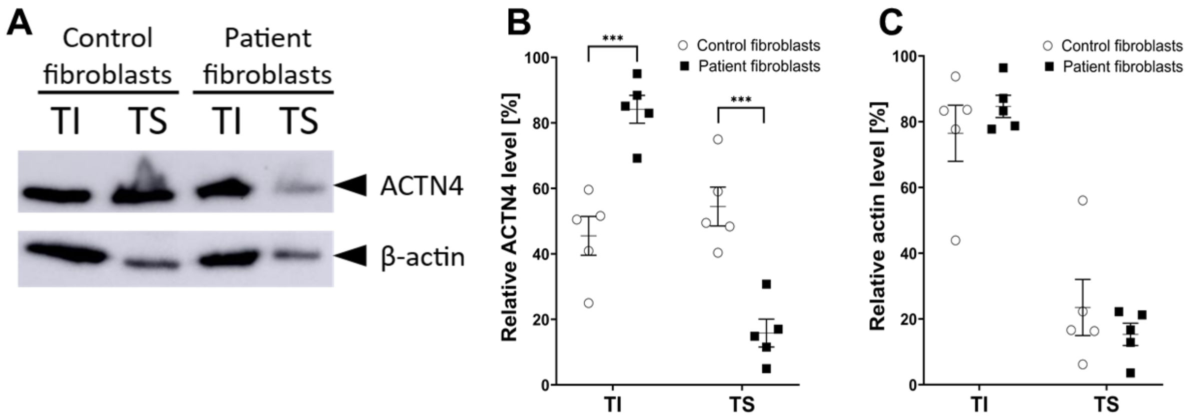

Supplementary Figure 3: ACTN4 p.(Ile150Met) forms Triton X-100 insoluble structures. Healthy control fibroblasts and patient fibroblasts were lysed in Triton X-100 containing lysis buffer and centrifuges to obtain Triton X-100 insoluble (TI) and soluble (TS) fractions. TI fractions contain large cytoskeletal structures, whereas TS fractions contain F- and G-actin. (**A**) Representative western blot results for fractionation experiments. (**B, C**) Densitometry of fractionation experiments. (**B**) A significantly higher expression level of ACTN4 in the TI fraction from patient fibroblasts (ACTN4 p.(Ile150Met)) compared to control fibroblasts (ACTN4 WT) is visible. (**C**) In contrast, the distribution of actin is comparable between control and patient fibroblasts. Results of n=5 experiments are displayed as relative expression levels (TS/[TI+TS] and TI/[TI+TS]) (Mean and SEM, *** P<0.001).

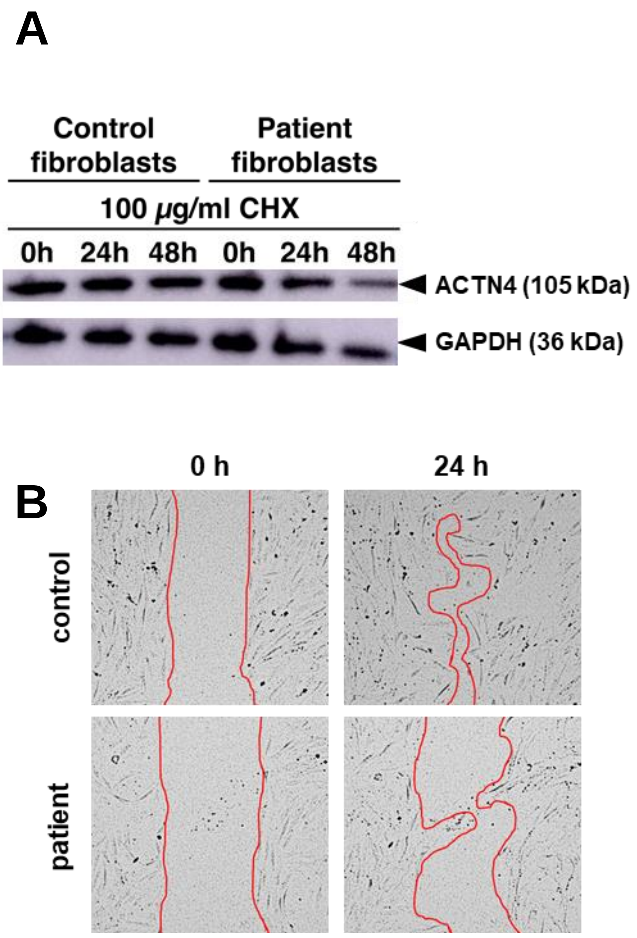

Supplementary Figure 4: ACTN4 p.(Ile150Met) is less stable than the WT form and leads to reduced motility in primary fibroblast. (**A**) Representative western blot of CHX pulse assay for control (ACTN4 WT) and patient (ACTN4 p.(Ile150Met)) fibroblasts. For the densitometry results of CHX pulse chase assay experiments please see Figure 1H in the manuscript. (**B**) Representative images for Figure 1I in the manuscript: Control and patient fibroblasts were plated in an ibidi 2-chamber silicone insert. Insert was removed and cell migration was monitored in real time.

Supplementary References

S1. Allison SJ. Benefits of actin cytoskeleton stabilization. *Nature Reviews Nephrology*. 2022/07/01 2022;18(7):413-413. doi:10.1038/s41581-022-00596-7

S2. Djinović-Carugo K, Young P, Gautel M, Saraste M. Molecular Basis for Cross-Linking of Actin Filaments: Structure of the α-Actinin Rod. *Cell*. 1999/08/20/ 1999;98(4):537-546. doi:10.1016/S0092-8674(00)81981-9

S3. Tang J, Taylor DW, Taylor KA. The three-dimensional structure of α-actinin obtained by cryoelectron microscopy suggests a model for Ca2+-dependent actin binding11Edited by W. Baumeister. *Journal of Molecular Biology*. 2001/07/20/ 2001;310(4):845-858. doi:10.1006/jmbi.2001.4789

S4. Amberger JS, Bocchini CA, Schiettecatte F, Scott AF, Hamosh A. OMIM.org: Online Mendelian Inheritance in Man (OMIM®), an online catalog of human genes and genetic disorders. *Nucleic Acids Research*. 2014;43(D1):D789-D798. doi:10.1093/nar/gku1205

S5. Drenckhahn D, Franke RP. Ultrastructural organization of contractile and cytoskeletal proteins in glomerular podocytes of chicken, rat, and man. *Laboratory investigation; a journal of technical methods and pathology*. 1988/11// 1988;59(5):673-682

S6. Dandapani SV, Sugimoto H, Matthews BD, et al. α-Actinin-4 Is Required for Normal Podocyte Adhesion. *Journal of Biological Chemistry*. 2007;282(1):467-477. doi:10.1074/jbc.M605024200

S7. Weins A, Kenlan P, Herbert S, et al. Mutational and biological analysis of α-actinin-4 in focal segmental glomerulosclerosis. Journal of the American Society of Nephrology2005. p. 3694-3701

S8. Choi HJ, Lee BH, Cho HY, et al. Familial Focal Segmental Glomerulosclerosis Associated With an ACTN4 Mutation and Paternal Germline Mosaicism. *American Journal of Kidney Diseases*. 2008/05/01/ 2008;51(5):834-838. doi:10.1053/j.ajkd.2008.01.018

S9. Gee HY, Sadowski CE, Aggarwal PK, et al. FAT1 mutations cause a glomerulotubular nephropathy. *Nature communications*. Feb 24 2016;7:10822. doi:10.1038/ncomms10822

S10. Gupta IR, Baldwin C, Auguste D, et al. ARHGDIA: a novel gene implicated in nephrotic syndrome. *J Med Genet*. May 2013;50(5):330-8. doi:10.1136/jmedgenet-2012-101442

S11. Slaats GG, Isabella CR, Kroes HY, et al. MKS1 regulates ciliary INPP5E levels in Joubert syndrome. *J Med Genet*. Jan 2016;53(1):62-72. doi:10.1136/jmedgenet-2015-103250

S12. Vélez-Ortega AC, Frolenkov GI. Building and repairing the stereocilia cytoskeleton in mammalian auditory hair cells. *Hear Res*. May 2019;376:47-57. doi:10.1016/j.heares.2018.12.012

S13. Karczewski KJ, Francioli LC, Tiao G, et al. The mutational constraint spectrum quantified from variation in 141,456 humans. *Nature*. 2020/05/01 2020;581(7809):434-443. doi:10.1038/s41586-020-2308-7

S14. Landrum MJ, Lee JM, Benson M, et al. ClinVar: improving access to variant interpretations and supporting evidence. *Nucleic Acids Research*. 2017;46(D1):D1062-D1067. doi:10.1093/nar/gkx1153

S15. Ng PC, Henikoff S. Predicting deleterious amino acid substitutions. *Genome Res*. May 2001;11(5):863-74. doi:10.1101/gr.176601

S16. Steinhaus R, Proft S, Schuelke M, Cooper DN, Schwarz Jana M, Seelow D. MutationTaster2021. *Nucleic Acids Research*. 2021;49(W1):W446-W451. doi:10.1093/nar/gkab266
S17. Adzhubei IA, Schmidt S, Peshkin L, et al. A method and server for predicting damaging missense mutations. *Nature Methods*. 2010/04/01 2010;7(4):248-249. doi:10.1038/nmeth0410-248
S18. Krtil J, Pláteník J, Kazderová M, Tesař V, Zima T. Culture Methods of Glomerular Podocytes. *Kidney and Blood Pressure Research*. 2007;30(3):162-174. doi:10.1159/000102520
S19. Podocytes in culture: Past, present, and future, 72 26-36 (2007). https://www.sciencedirect.com/science/article/pii/S0085253815525155?via%3Dihub
S20. Michaud JL, Hosseini-Abardeh M, Farah K, Kennedy CR. Modulating alpha-actinin-4 dynamics in podocytes. *Cell Motil Cytoskeleton*. Mar 2009;66(3):166-78. doi:10.1002/cm.20339
S21. Liem RK. Cytoskeletal Integrators: The Spectrin Superfamily. *Cold Spring Harb Perspect Biol*. Oct 3 2016;8(10)doi:10.1101/cshperspect.a018259
S22. Kao S-H, Wang W-L, Chen C-Y, et al. Analysis of Protein Stability by the Cycloheximide Chase Assay. *Bio-protocol*. 2015/01/05 2015;5(1):e1374. doi:10.21769/BioProtoc.1374
S23. Morishita H, Kawai K, Egami Y, Honda K, Araki N. Live-cell imaging and CLEM reveal the existence of ACTN4-dependent ruffle-edge lamellipodia acting as a novel mode of cell migration. *Exp Cell Res*. Sep 1 2024;442(2):114232. doi:10.1016/j.yexcr.2024.114232
S24. Richards S, Aziz N, Bale S, et al. Standards and guidelines for the interpretation of sequence variants: a joint consensus recommendation of the American College of Medical Genetics and Genomics and the Association for Molecular Pathology. *Genetics in Medicine*. 2015/05/01/ 2015;17(5):405-424. doi:10.1038/gim.2015.30
S25. Feng D, Notbohm J, Benjamin A, et al. Disease-causing mutation in α-actinin-4 promotes podocyte detachment through maladaptation to periodic stretch. *Proc Natl Acad Sci U S A*. Feb 13 2018;115(7):1517-1522. doi:10.1073/pnas.1717870115
S26. Tabrizi SJ, Flower MD, Ross CA, Wild EJ. Huntington disease: new insights into molecular pathogenesis and therapeutic opportunities. *Nat Rev Neurol*. Oct 2020;16(10):529-546. doi:10.1038/s41582-020-0389-4
S27. Horwich A. Protein aggregation in disease: a role for folding intermediates forming specific multimeric interactions. *The Journal of Clinical Investigation*. 11/01/ 2002;110(9):1221-1232. doi:10.1172/JCI16781
S28. Yao J, Le TC, Kos CH, et al. α-Actinin-4-Mediated FSGS: An Inherited Kidney Disease Caused by an Aggregated and Rapidly Degraded Cytoskeletal Protein. *PLOS Biology*. 2004;2(6):e167. doi:10.1371/journal.pbio.0020167
S29. Vangipuram M, Ting D, Kim S, Diaz R, Schüle B. Skin punch biopsy explant culture for derivation of primary human fibroblasts. Article. *Journal of visualized experiments : JoVE*. 2013;(77):e3779. doi:10.3791/3779
S30. Sherr CJ, DePinho RA. Cellular Senescence: Minireview Mitotic Clock or Culture Shock? *Cell*. 2000/08/18/ 2000;102(4):407-410. doi:10.1016/S0092-8674(00)00046-5
S31. Lewerentz J, Johansson A-M, Stenberg P. The path to immortalization of cells starts by managing stress through gene duplications. *Experimental Cell Research*. 2023/01/01/ 2023;422(1):113431. doi:10.1016/j.yexcr.2022.113431
S32. Waldera-Lupa DM, Kalfalah F, Florea AM, et al. Proteome-wide analysis reveals an age-associated cellular phenotype of in situ aged human fibroblasts. Article. *Aging*. 2014;6(10):856-878. doi:10.18632/aging.100698

S33. Peterson C, Goldman JE. Alterations in calcium content and biochemical processes in cultured skin fibroblasts from aged and Alzheimer donors. *Proceedings of the National Academy of Sciences*. 1986;83(8):2758-2762. doi:10.1073/pnas.83.8.2758
S34. Kálmán S, Garbett KA, Janka Z, Mirnics K. Human dermal fibroblasts in psychiatry research. *Neuroscience*. 2016/04/21/ 2016;320:105-121. doi:10.1016/j.neuroscience.2016.01.067
S35. Inoue K. CRISPR-activated patient fibroblasts for modeling of familial Alzheimer's disease. *Neuroscience Research*. 2021/11/01/ 2021;172:7-12. doi:10.1016/j.neures.2021.03.008
S36. Weins A, Schlondorff JS, Nakamura F, et al. Disease-associated mutant α-actinin-4 reveals a mechanism for regulating its F-actin-binding affinity. *Proceedings of the National Academy of Sciences*. 2007/10/09 2007;104(41):16080-16085. doi:10.1073/pnas.0702451104
S37. 14th Biennial International Podocyte Conference. Glomerular Diseases. 2023;3(Suppl. 1):1-98. doi:10.1159/000530913
S38 Hauer NN, Popp B, Schoeller E, et al. Clinical relevance of systematic phenotyping and exome sequencing in patients with short stature. *Genetics in medicine : official journal of the American College of Medical Genetics*. Jun 2018;20(6):630-638. doi:10.1038/gim.2017.159
S39. Ng PC, Henikoff S. Predicting deleterious amino acid substitutions. *Genome Res*. May 2001;11(5):863-74. doi:10.1101/gr.176601
S40. Steinhaus R, Proft S, Schuelke M, Cooper DN, Schwarz Jana M, Seelow D. MutationTaster2021. *Nucleic Acids Research*. 2021;49(W1):W446-W451. doi:10.1093/nar/gkab266
S41. Adzhubei IA, Schmidt S, Peshkin L, et al. A method and server for predicting damaging missense mutations. *Nature Methods*. 2010/04/01 2010;7(4):248-249. doi:10.1038/nmeth0410-248
S42. Tiosano D, Baris HN, Chen A, et al. Mutations in PIK3C2A cause syndromic short stature, skeletal abnormalities, and cataracts associated with ciliary dysfunction. *PLOS Genetics*. 2019;15(4):e1008088. doi:10.1371/journal.pgen.1008088
S43. Schindelin J, Arganda-Carreras I, Frise E, et al. Fiji: an open-source platform for biological-image analysis. *Nat Methods*. Jun 28 2012;9(7):676-82. doi:10.1038/nmeth.201
S44. Degasperi A, Birtwistle MR, Volinsky N, Rauch J, Kolch W, Kholodenko BN. Evaluating Strategies to Normalise Biological Replicates of Western Blot Data. *PLOS ONE*. 2014;9(1):e87293. doi:10.1371/journal.pone.0087293
S45. He Z, Wu K, Xie W, Chen J. Case report and literature review: A de novo pathogenic missense variant in ACTN4 gene caused rapid progression to end-stage renal disease. Case Report. *Frontiers in Pediatrics*. 2022-August-25 2022;10doi:10.3389/fped.2022.930258
